# *Brevibacterium paucivorans* bacteremia: case report and review of the literature

**DOI:** 10.1186/s12879-019-3962-y

**Published:** 2019-04-25

**Authors:** Nobuhiro Asai, Hiroyuki Suematsu, Atsuko Yamada, Hiroki Watanabe, Naoya Nishiyama, Daisuke Sakanashi, Hideo Kato, Arufumi Shiota, Mao Hagihara, Yusuke Koizumi, Yuka Yamagishi, Hiroshige Mikamo

**Affiliations:** 1 Department of Clinical Infectious Diseases, Aichi Medical University Hospital, ₸480-1195 1-1 Yazakokarimata, Nagakute, Aichi Japan; 2 Department of Infection Control and Prevention, Aichi Medical University Hospital, Nagakute, Japan

**Keywords:** Brevibacterium paucivorans, Compromised host, Bacteremia, Immunocompetent

## Abstract

**Background:**

Brevibacteria are obligate aerobic gram-positive rods that are associated with milk products and are also found on human skin. *Brevibacterium* has been reported as a rare cause of catheter related blood steam infection mainly in immunocompromised hosts such as malignancies or AIDS patients.

**Case presentation:**

A 94-year old woman, which had a past history of diabetes mellitus and chronic heart failure, presented with high fever associated with decreased oral intake and appetite loss and was admitted to our institute. A physical examination at the time of presentation was unremarkable. On day 2, both blood cultures collected on admission became positive with coryneform organism within 24 h without Staphylococci and *Brevibacterium species* were identified by Matrix-assisted laser desorption/ionization time-of-flight mass spectrometry. Subsequently, genetic investigation by 16S ribosomal RNA analysis was performed in order to identify the organism. Finally, the result identified this pathogen as *Brevibacterium paucivorans* with 99.5% homology on the Ez taxon database.

The patient was started empirically on meropenem and teicoplanin for broad-spectrum antibiotic coverage. The patient’s fever finally abated and labs were also improved. On day 14, the antibiotic therapy was discontinued. The site of infections was unknown. We hereby report a case of *Brevibacterium paicivorans* bacteremia in an immunocompetent patient and review cases of *Brevibacterium* specises bacteremia previously reported. This is the first case of *B. paucivorans* bacteremia as far as we could search.

**Conclusion:**

Physicians and microbiologists should be aware that Brevibacteria are uncommon but important agents which could cause opportunistic infections in immunocompetent.

## Background

*Brevibacterium* specises had been considered nonvirulent until infections mainly in immunocompromised patients were reported [1–3]. We report a case of *Brevibacterium paucivorans* bacteremia and review cases of *B. species* bacteremia which have been previously published. This is the first report of *Brevibacterium paucivorans* bacteremia in an elderly immunocompetent patient who was suffering from diabetes mellitus and chronic heart failure, as far as we could search.

## Case report

**Fig. 1:**
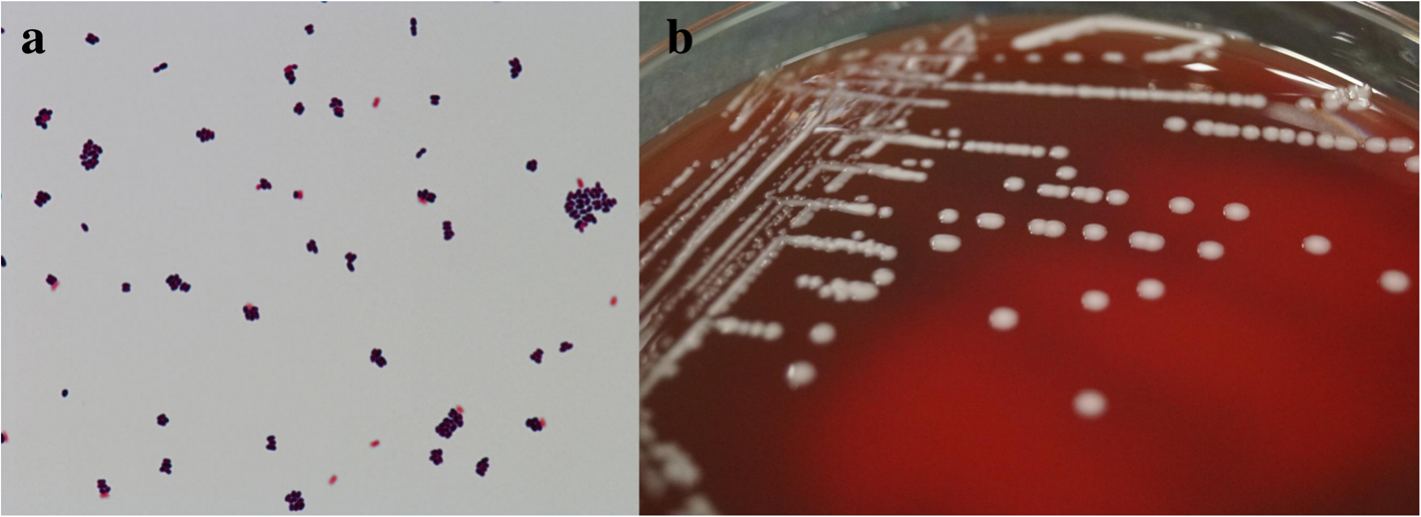
showed (**a**) blood culture Gram staining and (**b**) *Brevibacterium paucinovorans* colony morphology on trypticase soy agar II containing 5% sheep blood

A 94-year old woman presented with high fever associated with decreased oral intake and appetite loss and was admitted to our institute. She had been diagnosed as having diabetes mellitus, mild chronic kidney disease, chronic heart failure and stayed at a nursing home. She was a wheelchair-user. At the initial presentation, the patient had a body temperature of 40.2 °C, blood pressure of 183/81 mmHg, and pulse of 74 beats per min. Hypoxemia was not confirmed. The physical examination was unremarkable. Chest X-ray and urine test were normal. Laboratory tests revealed an elevation of blood urea nitrogen 23.8 mg/dl, creatinine 1.14 mg/dl and C-reactive protein 1.93 mg/dl. Platelet count was low at 105,000/μl. White cell count, hemoglobin and liver function tests were within normal range as shown in supplementary file. Two sets of blood cultures for aerobic and anaerobic bacteria, mycobacteria and fungi were drawn. Then, the patient was started empirically on meropenem and teicoplanin for broad-spectrum antibiotic coverage. In addition to blood cultures, a urinalysis with culture and a chest X-ray and CT were performed and found to be normal. The patient had no clinically evident sites of infection by history or physical examination. On day 2, a coryneform organism was recovered for 32 h by BACTEC (BD, Tokyo, Japan) from both the aerobic and anaerobic tubes of all blood cultures. *Brevibacterium species* were identified by Matrix-assisted laser desorption/ionization time-of-flight mass spectrometry (MALDI-TOF MS). The score value was 2.36. On gram-stained smears from the culture plates, the organisms appeared as Gram-positive, club-shaped, slightly curved rods, and some coccal forms were present (Fig. 1a). The bacteria were subcultured on Trypticase Soy Agar II with 5% Sheep Blood (BD, Tokyo, Japan) at 35 °C in 5% CO_2_, which resulted in a gray-white, smooth, non-hemolytic colonies after a 48-incubation (Fig. 1b). Subsequently, genetic investigation by 16S ribosomal RNA analysis was performed in order to identify the organism. Finally, the result identified this pathogen as *Brevibacterium paucivorans* with 99.5% homology on the Ez taxon database (http://www.ezbiocloud.net/eztaxon).

**Fig. 2:**
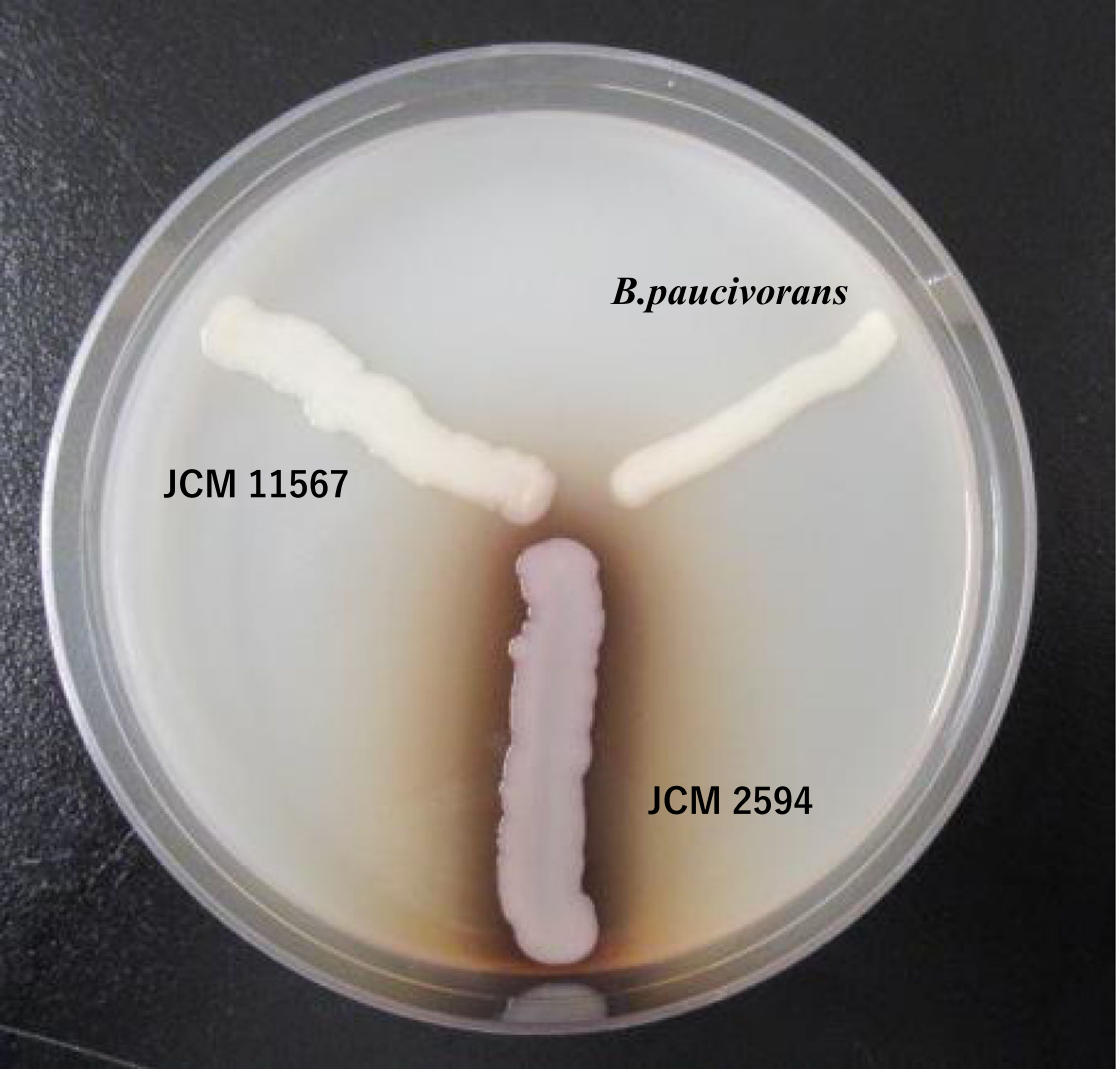
showed that both the organism, *B. paucivorans* and JCM 11567^T^ had a lack of hydrolysis of casein, while JCM 2594^T^ showed a hydrolysis of casein

For comparison of a hydrolysis of casein in the organism, we obtained a type strain of *B. casei*, JCM 2594^T^ and of *B. paucivorans*, JCM 11567^T^, from the Japan Collection of Microorganisms (JCM). Pyrazinamidase test was performed using PZA broth (Kyokuto Pharmaceutical Inc., Tokyo, Japan). Casein hydrolysis test was performed as follows. 1) inoculate the organism on a skim milk agar, 2) incubate the plate at 37 °C, 3) examine the plate for zone of hydrolysis following incubation. Both the organism and JCM 11567^T^ showed a lack of hydrolysis of casein, while a hydrolysis of casein was confirmed in JCM 2594^T^ as shown in Fig. 2. The organism had an absence of pyrazinamidase, while JCM 2594^T^ showed a presence of pyrazinamidase. Additional microbiological tests by API 50CH showed that utilization of D-arabinose and gluconate was negative. These results were consistent with the organism as *B. paucivorans*. Antimicrobial susceptibility testing revealed that the organism was susceptible to MEPM. Although the peripheral venous catheter site showed no erythema or tenderness, the catheter was removed without culture, and a follow-up blood culture remained negative after therapy lasting for 7 days. The patient’s fever finally abated and labs were also improved. On day 14, the antibiotic therapy was discontinued. On day 28 from admission, fever recurred and blood cultures were performed. *Candida parapsilosis* was isolated by 2 sets of blood cultures, and she was diagnosed as having candidemia. While L-AMB was started for *Candida parapsilosis* bacteremia, she died by candidemia on day 35.

Antimicrobial susceptibility testing was performed for the strain using the broth microdilution method (Dry Plate^®^, Eiken Chemical co., Ltd., Tokyo, Japan) according to the Clinical and Laboratory Standards Institute guidelines [4]. The isolate was susceptible to gentamicin [minimum inhibitory concentration (MIC) = 1 μg/ml], ciplofloxacin (MIC = 0.25 μg/ml), vancomycin (MIC≦0.5 μg/ml), meropenem (MIC≦0.5 μg/ml) and rifampicin (MIC≦0.12 μg/ml), and was resistant to clindamycin (MIC> 4 μg/ml), and was intermediately resistant to ceftriaxone (MIC = 2 μg/ml),and cefepim (MIC = 2 μg/ml) as shown in Table 1.

**Table 1:** Antimicrobial susceptibility of *Brevibacterium paucivorans* isolated from blood culture

Antimicrobial agents	MIC (μg/mL)	Interpretation
Ampicillin	> 0.5	
Gentamicin	1	S
Amikacin	8	
Ceftazidime	<=1	
Ceftriaxone	2	I
Cefazolin	4	
Cefepime	2	I
Clindamycin	> 4	R
Ciprofloxacin	0.25	S
Imipenem	<=0.5	S
Levofloxacin	<=0.25	
Rifampicin	<=0.12	S
Vancomycin	<=0.5	S
Daptomycin	0.5	S
Meropenem	<=0.5	S
Doxycycline	<=0.5	S

*MIC* minimum inhibitory concentration, *S* susceptible, I intermediate, *R* resistant

## Discussion

*Brevibacterium* species are gram-positive, irregular, slender, rod-shaped, non-acid fast bacteria which resemble corynebacteria. At the present time, ten species are classified in this genus: *B. linens, B. iodinum, B. epidermidis, B. casei, B. mcbrellneri, B. otitidis, B. avium, B. paucivorans, B. luteolum and B. sanguinis* [4–7]. The main habitats of *Brevibacterium* sp. are dairy products, where the bacteria contribute to the aroma and color. They are also found on human skin surfaces, genital hair and otorrhea [6, 8, 9]. Twelve bacteremia cases caused by *Brevibacterium* species including ours have been previously reported as shown in Table 2 [1–4, 10–15]. Five and 2 had hematologic or non-hematologic malignancies, and acquired immunodeficiency syndrome (AIDS) of the 12 patients, respectively. Five of the 12 (42%) had malignancies, and 2 of the 12 (17%) had AIDS. Ten of the 11 (91%) for which information is available had indwelling central venous catheter (CVC), including CV port. Four of the 12 were not conventional immunocompromised patients, but those who suffered from severe diseases. As for outcome, 10 of 11 (91%) patients whose information regarding the outcome is available were improved. However, 3 of the 10 (30%) patients recurred after antibiotic therapy from 13 to 28 days. These results suggest that *Brevibacterium* bacteremia could have a poor prognosis the same as gram-negative rods bacteremia. To underestimate these unspecific but relevant clinical symptoms and misinterpretation as apathogenic organisms could contribute result in delayed diagnosis and treatment of this emerging and mainly opportunistic pathogen. Thus, it is important to sensitize clinicians and microbiologists to this environmental pathogenic microorganism.

**Table 2:** Previous reports about *Brevibacterium* species bacteremia

Author (Year)	Sex	Age	*Brevibacterium* species	Underlying disease	Indwelling catheter	Treatment regimen	Outcome
McCaughey (1991) [1]	M	40	*Epidermidis*	Zollinger-Ellison syndrome	+	EM	Improved
Lina (1994)	M	19	Not specified	Lymphoblastic lymphoma	+	TEIC AMK	Recurred
Reinert (1995) [3]	M	25	*Casei*	Testicular chorion carcinoma	+	PIPC TEIC	Recurred
Kaukoranta-Tolvanen (1995) [10]	F	46	*Casei*	Non-Hodgkin Lymphoma	+	ND	Recurred
Castagnola (1997) [11]			*Casei*	Neuro-blastoma	+	ND	Improved
Brazzola (2000) [12]	F	18	*Casei*	AIDS	+	CPFX	Improved
Ogunc (2002) [13]	ND	60	Not specified	Chronic lymphatic leukemia	ND	CAZ AMK	Improved
Janda (2003) [4]	M	34	*Casei*	AIDS	+	VCM GM	Improved
Beukinga (2004) [14]	F	43	*Casei*	Crohn’s disease	+	VCM	Dead
Beukinga (2004) [14]	M	31	*Casei*	Received HD. Disease was not mentioned	+	VCM	Improved
Ulrich (2006) [15]	F	62	*Casei*	Pulmonary hypertension	+	VCM followed by MFLX	Improved
Ours (2018)	F	94	*Paucivorans*	Diabetes mellitus Chronic heart failure	–	MEPM TEIC	Improved^a^

*M* male, *F* female, *ND* not described, *AIDS* acquired immunodeficiency syndrome, *EM* erythromycin, *TEIC* teicoplanin, *AMK* amikacin, *PIPC* piperacillin, *CPFX* ciplofloxacin, *VCM* vancomycin, *GM* gentamicin, *MFLX* moxifloxacin, *MEPM* meropenem. ^a^Death caused others

As for the infection site, considering the clinical course and bacteria of gram-positive rods, it is possible that this case might be caused by catheter-related blood stream infection (CRBSI). Unfortunately, it was unclear, because a catheter culture was not obtained.

The patient died of candidemia. It might have been caused by CRBSI or bowel translocation. This patient was a particularly difficult case to manage due to multiple comorbidities such as chronic heart failure and mild chronic kidney disease. In addition, she was super-elderly and a wheelchair user. Both the poor general condition and the clinical course including antibiotic therapy might have contributed to her candidemia.

## Conclusion

Physicians and microbiologists should be aware that Brevibacteria are uncommon but important agents which could cause opportunistic infections in immunocompetent as well as immunocompromised patients.

## Abbreviations

AIDS: Acquired immunodeficiency syndrome
CRBSI: Catheter-related blood stream infection
CT: Computed tomography
CVC: Central venous catheter
JCM: the Japan collection of microorganisms
MALDI-TOF-MS: Matrix-assisted laser desortion ionization time of light
MEPM: Meropenem
MIC: Minimum inhibitory concentration
